# Adaptation and Psychometric Properties of the Spanish Version of Child and Youth Resilience Measure (CYRM-32)

**DOI:** 10.3389/fpsyg.2019.01410

**Published:** 2019-06-28

**Authors:** María Llistosella, Teresa Gutiérrez-Rosado, Rocío Rodríguez-Rey, Linda Liebenberg, Ángela Bejarano, Juana Gómez-Benito, Joaquín T. Limonero

**Affiliations:** ^1^Center of Primary Health Care Terrassa Nord, Consorci Sanitari de Terrassa (CST), Barcelona, Spain; ^2^Department of Clinical and Health Psychology, Faculty of Psychology, Autonomous University of Barcelona, Barcelona, Spain; ^3^Faculty of Health and Biomedical Sciences, Universidad Europea de Madrid, Madrid, Spain; ^4^Faculty of Graduate Studies, Dalhousie University, Halifax, NS, Canada; ^5^Secretariat for Social Integration, Sub-Directorate for Matters LGBT, Bogotá, Colombia; ^6^Department of Social Psychology and Quantitative Psychology, Faculty of Psychology, University of Barcelona, Barcelona, Spain; ^7^Stress and Health Research Group (GIES), Faculty of Psychology, Autonomous University of Barcelona, Barcelona, Spain

**Keywords:** resilience, at-risk young, social exclusion, reliability, validity, mixed-methods, cultural adaptation

## Abstract

Resilience is defined as a dynamic process that entails a positive adaptation to contexts of adversity. According to the ecological model, resilient behavior emerges as a result of the interaction between individual, relational, community and cultural variables. The Child and Youth Resilience Measure (CYRM-28), developed in Canada and based on the ecological model, has been validated in several countries. The objective of this article is to present the cultural adaptation (studies I and II) and validation (study III) in Spanish at risk youth. A three-study mixed-method design was selected. Study I includes translations and a confirmatory and exploratory factor analysis of a sample of 270 Spanish young persons (56.9% boys) aged between 12 and 18 years (*M* = 14.65; SD = 1.27) from an urban public elementary school. Study II uses semi-structured interviews with adolescents identified as resilient and presents a content analysis and a reformulation of items with experts. Study III includes the confirmatory factor analysis, internal consistency, test–retest, convergent and discriminant validity, and multivariate analysis of variance to explore group differences of the resulting scale CYRM-32. The sample consisted of 432 at-risk young persons (54.9% boys) aged between 12 and 19 years old (*M* = 14.99; SD = 2.23). The results confirm the adequate psychometric properties of the CYRM-32 scale. From the original scale, 4 items were eliminated, 5 were reformulated presenting very low saturations. Meanwhile, 6 items were added to the cultural adaptation phase, resulting in a 32-item scale. The confirmatory analysis confirms the 3 factors expected in the CYRM-32 scale with good reliability indexes (Cronbach’s α total scale 0.88, family interaction 0.79, interaction with others 0.72 and individual skills 0.78). The scale has convergent and discriminant validity in relation to the Brief Resilient Coping Scale, Coping Scale for Adolescents and Self-Concept. Significant differences were found in the scores of the CYRM-32 scale for the ethnic variable [*F*(71. 358) = 1.714, *p* < 0.001], while no differences appear according to age and gender. This finding confirms the importance of culture in the resiliency processes. The CYRM-32 scale has good psychometric properties and is a new alternative for measuring resilience in Spanish at-risk youth.

## Introduction

Resilience is a construct used to explain the processes that result in good outcomes despite high-risk situations that pose a threat to positive adaptation and self-development (Masten and Coatsworth, 1998; Masten, 1999, 2007; Masten et al., 1999). Definitions of what constitutes appropriate adaptation however, varies in relation to the cultural, historical and/or social context (Masten and Coatsworth, 1998). Thus, currently there is no a universal definition of resilience (Aburn et al., 2016), but the literature show the influence of the contextual factors in the development of resilience and coping among youth (Clauss-Ehlers, 2008). Moreover, sociocultural aspects of support can promote and contribute to resilience.

For this reason, transcultural research is needed for studying resilience and to determine what can be considered as positive development in different socio-cultural contexts. Masten (1999) has proposed the following questions: “Should successful development be defined only within cultural context? What happens when subcultural norms differ from the majority culture?” (p. 283).

From a contextual and ecological perspective, resilience has been defined by Ungar (2008) as “in the context of exposure to significant adversity, whether psychological, environmental, or both, resilience is both the capacity of the individuals to navigate their way to health-sustaining resources, including opportunities to experience feelings of wellbeing, and a condition of the individual’s family, community and culture to provide these health resources in culturally meaningful ways” (p. 225). From this point of view, the resilience process of the child should be understood in relation to the context; it relates to the threats that people have to face and the interactions between risk exposure and the available resources that permit adaptation to the environmental and personal challenges (Ungar et al., 2008). Boyden and Mann (2005) as well as Ungar (2004) have argued that the way we understand resilience is negotiated discursively and influenced by the culture and social context in which it is located. Consequently, it is necessary to continue studying the underlying mechanisms and processes of resilience across different cultures (Boyden and Mann, 2005).

Despite the importance of culture in the development of resilience, many instruments used to measure resilience have neglected these cultural factors (Clauss-Ehlers, 2008).

In this line, according to Ungar (2011), in the measurement of resilience the effects of cultural immersion in dominant cultures and heterogeneity in ethno-racial minorities have been overlooked. This is important because the identification of resilience factors that contribute to the process of adaptation to adversity contribute to the improvement of intervention programs that empower youth to manage the resources that sustain their wellbeing (Ungar, 2004, 2008).

For this reason, it is necessary to have evaluation tools based on an overall theoretical framework of resilience. Several resilience measures have been developed and validated for the use of children and young people. These include Youth Resiliency: Assessing Developmental Strengths (YR:ADS), validated in Canada (Donnon and Hammond, 2007); The Resilience Scale for Adolescents (READ), validated in Norway, Ireland, Mexico, and Italy (Hjemdal et al., 2006; Von Soest et al., 2010; Stratta et al., 2012; Ruvalcaba-Romero et al., 2015; Kelly et al., 2016), and the Child and Youth Resilience Measure (CYRM-28), validated in Canada, New Zealand, and Iran (Ungar and Liebenberg, 2011; Liebenberg et al., 2012; Daigneault et al., 2013; Sanders et al., 2015; Kazerooni et al., 2016).

Additionally, measures that assess resilience-related constructs have been adapted for use with Spanish children and adolescents. For example, the Adolescent Coping Scale (Frydenberg and Lewis, 1996), the Coping Response Inventory for Youth (Forns et al., 2005) and The Adolescent Resilience Questionnaire (Guilera et al., 2015).

Given the need for resilience measures to be adapted to the different cultures, in addition to validation of psychometric properties, in this article we continue with the validation and adaptation of the CYRM-28. We focus on the CYRM-28, reflecting Bronfenbrenner’s Ecological Model (Bronfenbrenner, 1979). Additionally, the CYRM-28 was specifically developed to assess resilience in vulnerable adolescents across cultures and contexts (Ungar and Liebenberg, 2011). In their assessment of resilience measures, Windle et al. (2011) found the CYRM-28 to have high content validity and they presented this test as one of the few resilience measures that assesses multiple dimensions of resilience (culture, community, relationship and individual) and shows conceptual adequacy. Initially the CYRM-28 included 14 sites around the world. Later, following this work, the measure has been validated for use in other countries, as we have indicated previously, but not yet in Spain.

**FIGURE 1:**
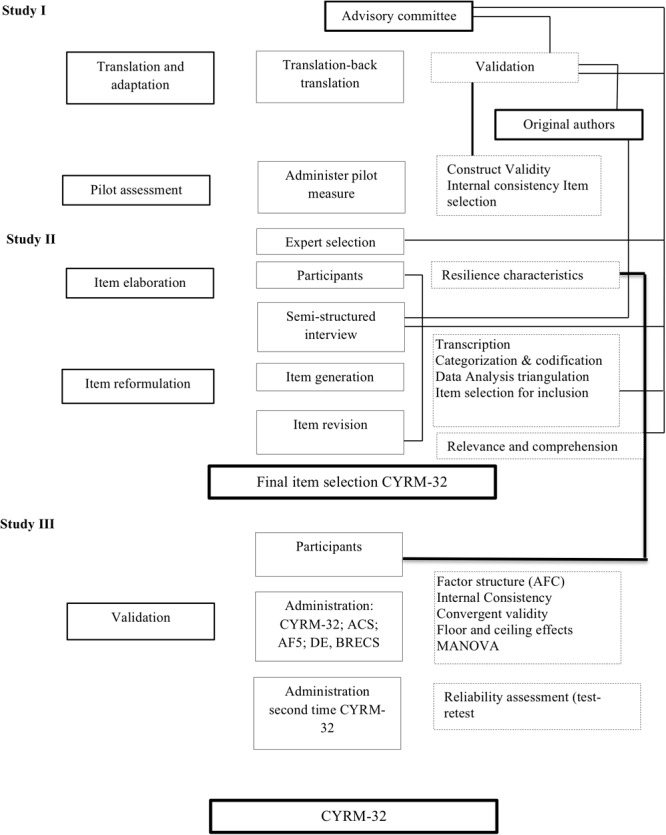
Procedure flow.

This work presents the cultural adaptation and validation of the Child and Youth Resilience Measure (CYRM-32) to a Spanish sample of risk youth. It includes three studies: (1) the exploration of the psychometric properties of the original scale (CYRM-28) after being translated to the Spanish language, (2) the development of new items for the CYRM adapted to the local context and the reformulation of conflicting items, and (3) the validation of the final CYRM-32 adapted scale (Figure 1).

We decided to use a mixed methods design was in order to adapt the CYRM-28 scale (Ungar and Liebenberg, 2011; Liebenberg et al., 2012). The relevance of mixed methods to enable the understanding of common and unique aspects of resilience across cultural has been discussed during the development of the original scale (Ungar and Liebenberg, 2011). The mixed methods used here in our study allowed us to compare the results of our quantitative findings with the experiences of young people at risk and the individual and cultural resources that intervened in their resilient processes (Ungar and Liebenberg, 2011).

The study was approved by the Ethics Committee Institutional Review Board of the Consorci Sanitari de Terrassa (Spain). For all the studies, informed consent was obtained prior to data collection, from the high schools’ headmasters and participants.

## Study I

The purpose of this study was the translation of the CYRM-28 scale and a review of its factor structure using exploratory and confirmatory factor analyses.

### Participants

An evaluation committee of five professionals with knowledge about children at risk of social exclusion was formed. Specifically, the focus was on youth experiencing increased disconnection from their communities together with a loss of personal and social ties, and their families. This disconnection hinders access to opportunities and resources made available to support individuals living in low socioeconomic contexts and or who have recently experienced immigration.

A total of 270 young people in a high risk of social exclusion were randomly selected from two schools situated in a vulnerable neighborhood. Each year group consisted of four different classes or subgroups (30–35 students each) and two classes per school year (60–70 students) were randomly selected. The average age of the participants was 14.5 years (SD = 1.27, range 12.35 to 18.08); 56.9% were boys and 43.1% girls; 76% lived with both parents, 19% with a single mother and 2% with other people; 75.8% were European, 10.7% Latin American and 8.3% belonged to different ethnic groups.

### Instruments

In the first study, the original version of the Child and Youth Resilience Measure-28 (CYRM-28) (Ungar and Liebenberg, 2011) was used. It was designed as a screening tool to explore the resources (individual, relational, communal and cultural) available to youth aged 12 to 23 years, that may bolster their resilience processes (Ungar and Liebenberg, 2011; Liebenberg et al., 2012). The CYRM-28 has 28 items scored on a 5-point Likert scale (1 = not at all to 5 = a lot), and was designed as a self-reported measure which takes approximately 20 min to complete (Ungar and Liebenberg, 2011; Liebenberg et al., 2012). The Cronbach’s α of the three components of the scale showed the following values for each dimension: individual (α = 0.80); relational (α = 0.83); and contextual (α = 0.79) (Liebenberg et al., 2012).

### Data Analysis and Procedure

Following the guidelines provided by the original authors and the International Test Commission in construction and adaptation areas the back translation method was used to translate the CYRM-28 into Spanish (Beaton et al., 2000; Hambleton and John, 2003). Cycles of corrections and revisions were carried out until a definitive version was obtained for each item.

A confirmatory factor analysis (CFA) using AMOS version 22 was conducted on data gathered from the first youth sample, to explore whether the original factor structure proposed by Liebenberg et al. (2012) fitted our sample. In order to assess model fit, absolute fit indexes (χ^2^, χ^2^/*df*), relative fit indexes (IFI) and non-centrality fit indexes (CFI, RMSEA, SRMR) were used, as well as criteria for acceptable fit based on the degree of adjustment described by Hair (2010) (ratio χ^2^/*df* < 5; SRMR < 0.08; RMSEA < 0.08; GFI, CFI and IFI > 0.90). As the previously tested model failed to fit our data, an exploratory factor analysis (EFA) was conducted to ascertain the factor structure of the CRYM-28 with a confirmatory approach (Pett et al., 2003; Cumming et al., 2005). A confirmation of the adequacy of the data by means of the Kaiser-Meyer-Olkin test and the Bartlett’s test of sphericity was carried out (Costello and Osborne, 2005). The selection of factors was based on psychometric guidelines and Screen Plot or Screen Test of Cattell (Kline, 1994); coefficients greater than 0.40 and based on Kaiser’s criterion of eigenvalue equal or greater than 1.0, were considered (Kline, 1994; Nunnally and Bernstein, 1994). Internal consistency was obtained through Cronbach’s α (Cronbach, 1951).

### Results

#### Confirmatory Factor Analysis (CFA)

Results of the CFA show that the three-factor model tested did not provide an acceptable fit to our data. The chi-square statistic was significant, probably due to the sample size (Hair, 2010), the ratio (χ^2^/*df* = 2.89 < 5) was well within the limits that allowed the model to be accepted, RMSEA and SRMR values were acceptable (<0.08), but the CFI (0.61), IFI (0.62) and GFI (0.80) were below the level of acceptance (all of them < 0.90).

#### Exploratory Factor Analysis (EFA)

The Kaiser-Meyer-Olkin Measure of Sampling Adequacy was 0.73 middling but sufficient (Hair et al., 2014) and the Bartlett’s test of sphericity was appropriate (*X*^2^(378) = 1446,563; *p* = 0.000). Following the process of Ungar and Liebenberg (2011), EFA was carried out using Oblimin with Kaiser Rotation with three factors. Three components were identified that explained 30.8% of the total variance. Factor 1 “Family interaction,” explained 17.7% of the variance; factor 2 “Interaction with others” explained 7.17%; and, factor 3 “Individual skills,” 6% (see Table 1).

**Table 1:** Summary of factor loadings for principal component analysis for Oblimin three factor solution of the CYRM-28 (Spanish version).

Items components	1 Family interaction	2 Interaction with others	3 Individual skills	Liebenberg et al., 2012	Daigneault et al., 2013
17. My family stands by me during difficult times. (*Mi familia me apoya en los momentos difíciles*)	0.731			Caregiver	Familial
24. I feel safe when I am with my family/caregiver(s). (*Me siento a salvo junto a mis padres o tutores*)	0.721			Caregiver	Familial
6. Mis padres o tutores lo saben todo sobre mí. (*My parent*(*s*)*/caregiver*(*s*) *know a lot about me*)	0.610			Caregiver	Familial
26. I enjoy my family’s/caregiver’s cultural and family traditions. (*Disfruto de las tradiciones familiares con mis padres o tutores*)	0.547			Caregiver	Familial
12. I talk to my family/caregiver(s) about how I feel. (*Hablo sobre cómo me siento con mi familia o tutores legales*)	0.486			Caregiver	Familial
27. I enjoy my community’s traditions. (*Disfruto de las tradiciones de mi comunidad*)	0.448			Context	Community
15. I know where to go in my community to get help. (*Sé dónde acudir dentro de mi comunidad, cuando tengo algún problema*)	0.431			Individual	Individual
**23. I think it is important to help out in my community**. (*Creo que es importante ayudar en mi comunidad*)	0.401		0.314	Context	Community
28. I am proud to be Spanish? (*Estoy orgulloso de ser ciudadano de España?*)				Context	–
14. I feel supported by my friends. (*Mis amigos me apoyan*)		–0.774		Individual	Individual
18. My friends stand by me during difficult times. (*Mis amigos me apoyan en los momentos difíciles*)		–0.665		Individual	Individual
11. People think that I am fun to be with. (*La gente piensa que soy una persona divertida*)		–0.562		Individual	Individual
7. If I am hungry, there is enough to eat. (*Si tengo hambre, siempre hay suficiente comida para alimentarme*)		–0.480		Caregiver	Familial
**19. I am treated fairly in my community**. (*Soy tratado con igualdad dentro de mi comunidad*)	0.316	–0.458		Context	Individual
16. I feel I belong at my school. (*Siento que formo parte de mi escuela*)		–0.403		Context	Individual
22. I participate in organized religious activities. (*Participo en diversas actividades religiosas*)		0.369		Context	Community
2. I cooperate with people around me. (*Coopero con las personas de mi alrededor*)				Individual	Individual
10. I am proud of my ethnic background. (*Me siento orgulloso de mi origen étnico*)				Context	Community
5. Do you feel that your parent (s) watch you closely. (*Me siento vigilado por mis padres o tutores*)				Caregiver	–
21. I am aware of my own strengths. (*Soy consciente de mis puntos fuertes*)			0.717	Individual	Individual
20. I have opportunities to show others that I am becoming an adult and can act responsibly. (*Puedo demostrar a los demás que soy una persona adulta y responsable*)			0.557	Individual	Individual
4. I know how to behave in different social situations. (*Sé comportarme teniendo en cuenta las normas sociales*)			0.529	Individual	Individual
25. I have opportunities to develop skills that will be useful later in life (like job skills and skills to care for others. (*Tengo la oportunidad de desarrollar habilidades que me serán útiles en el futuro* (*Habilidades relacionadas con un oficio y habilidades sociales*)			0.506	Individual	Community
8. I try to finish what I start. (*Intento finalizar todo lo que empiezo*)			0.487	Individual	Individual
13. I am able to solve problems without harming myself or others (for example by using drugs and/or being violent. (*Puedo solucionar mis problemas sin hacerme daño ni hacer daño a terceras personas* (*por ejemplo sin caer en adicciones como la droga y sin usar la violencia*)			0.442	Individual	Familial
1. I have people I look up to. (*Conozco personas a las que admiro*)			0.395	Context	–
3. Getting an education is important to me. (*Tener una educación es importante para mí*)			0.380	Context	–
9. Spiritual beliefs are a source of strength for me. (*Mi fe me da fuerzas*)			0.366	Context	Community
**Cronbach’s α 0.783**	**0.720**	**0.506**	**0.622**		

*Bold items indicate factor loadings on more than one factor.*

Four of the 28 initial items (items 2, 5, 10, and 28) failed to load on any of the three factors, and two were identified (19, 23) as conflicting items. As such these items were removed from the scale. A further six more items were removed due to inadequate loadings [items 1 (0.395), 3 (0.380), 9 (0.366) and 22 (0.369)], and conflicting loadings (loadings > 0.3 in more than one factor; items 19, 23). Cronbach’s α were: Family interaction (0.720), Interaction with others (0.506), Individual skills (0.622) and for the totality of scale (0.783).

## Study II

The second study describes the cultural adaptation of the CRYM into the Spanish context, using qualitative interviews with adolescents identified as resilient which were carried out by experts.

### Participants

The second study included youth and resilience expert’s adults. Six young people (2 girls and 4 boys, aged 17–19) were purposively selected to participate. The participants were chosen by two experts from institutions that work with vulnerable populations, children, youth and families in high risk situations (i.e., experiencing poverty, low socioeconomic status, social exclusion, exposure to violence and social dislocation for example through immigration). The participants were identified as resilient (i.e., young people that despite having gone through adverse situations have a healthy lifestyle, positive social relationships and positive individual skills). Five professional (psychology, philology, social work and education; 1 male and 4 females), resilience experts participated. They were invited given their expertise in the conceptualization of the resilience in Spain and to explore and formulation the items.

Additionally, four randomly selected participants (2 girls and 2 boys, age 14–17 years old) from the first phase who had previously completed the CYRM-28 scale were also invited to study 2 to review the translated items that showed low loadings in the first phase of the study.

### Procedure

The second study included two phases (see Figure 1). In the first phase, two semi-structured individual interviews were conducted with the 6 young people and the 5 experts. These interviews were intended to generate new culturally and contextually relevant items for inclusion in the validated Spanish version of the CYRM-28. Accordingly, the interview guide used in the original development of the measure (Resilience Research Centre, 2009), was used in these interviews. All interviews were recorded and transcribed. Content analysis, conducted by two peer researchers, was used to identify themes reflecting culturally and contextually relevant resilience mechanisms and processes, for peer researchers. Identification of themes was guided by the original CYRM-28 factors (individual, caregivers and context). Emerging themes were used to create new items relevant to the local context on the basis of experts’ consensus.

The second phase of study two was intended to ensure the rigor of the new items fort this, data was triangulated. First, the five experts reviewed each of the reformulated and new items. Each item was assessed for relevance and comprehension using a 5-point Likert scale (1 = not at all relevant or comprehensive to 5 = extremely relevant or comprehensive). Additionally, a semi-structured interview was conducted with the small group of four youth from the original youth sample, to explore the comprehension and possible ambiguous aspects of the items with low loadings. The interviews were transcribed and a discourse analysis was undertaken in order to ensure that the perspectives each participant gave to issues related with the reviewed items was adequately captured.

### Results

#### New Items

The results were based on the narratives of the participants and the categories established in the CYRM-28. The emergent themes are presented according to the conceptual framework of the first Spanish version of the CYRM-28. For the three factors, 12 new items were generated and nine of them were included (see Table 2).

**Table 2:** Generation of new items.

Dimension	Coding theme	New item	Quotations
**Individual Factor**	Self-awareness	^∗^When I face a problem, I’m aware of my emotions and act according to how I feel in that moment. *Ante algún problema soy consciente de mis emociones y actúo según como me siento en el momento.*	“Sometimes I stay calm, think about what is going on with myself and think about how to solve the situation.” (Participant B) “Recognizing her hatred was a protective factor, because during a time she defended herself from the pain that she was feeling, and accepting that her father was an abuser. All those years she had a yearning of having a father by her side…but she start accepting that she didn’t love him and she felt hurt. The acceptance of this situation let her protect herself.” (Expert 1)
	Sense of humor	^∗^Despite difficulties, I usually smile. I think that I have good sense of humor. *A pesar de las dificultades suelo sonreír. Me considero una persona con buen sentido del humor.*	“Even if I’m sad I have to laugh. Laugh about me and everything, because if not I would be crying the whole day and that is not good at all.” (Participant B) “She has developed the good sense of humor by learning; as a positive reinforcement. She has a brother that doesn’t smile, and then she started to discover that smiling, people pay more attention to her than to her brother.” (Expert 5)
	Vision of the future	^∗^My strength helps me to go on and achieve my goals *Mi fortaleza me ayuda a seguir adelante y alcanzar Mis objetivos.*	“I think that going on with my life and not get desperate is good. Have the strength to keep going…” (Participant A) “If they talk with their family and agree, they can keep going as champions. It has to do with motivation and remember their purpose of life…Especially a tremendous will to move forward.” (Expert 4)
		^∗^I have aspirations and a clear and realistic vision of the future *Tengo aspiraciones y una visión de futuro clara y realista.*	“I belief and I want to finish high school and study in a university…I think that going on with my life and not get desperate is good. Have the strength to keep going…” (Participant A) “Having a realistic optimism. I mean, they can think that everything can have a good result, they have a positive vision of the future and think that they can control the course of their lives, but is important to have a good sense of reality and don’t let go over fantasies.” (Expert 2)
	Adaptation	^∗^I’m able to adapt to changes. *Soy capaz de adaptarme a los cambios.*	“Now that I’m here… I want to adapt where my family has brought me. I try to adapt to everything; to people, neighbors, everyone, to the school… I try to adapt little by little to everything.” (Participant A) “Maybe during a time I get myself isolated… and then I think that maybe that change can be good for me o for another person… and I try to adapt to the situation.” (Participant C)
	Autonomy	^∗^I usually make my own decisions and don’t let myself be influenced by others *Tiendo a tomar mis propias decisiones y no me dejo llevar por los demás.*	“A person can move on by itself, I mean without relying on anyone, that one’s objectives can be achieved by oneself no matter what.” (Participant C) “When I say that they have to have their head well set, I mean that they have to be responsible; basic competencies, a kid that worries about himself; an autonomous kid.” (Expert 4)
**Interaction with others**	Support to others	^∗^I support my peers. *Doy apoyo a mis compañeros.*	“More than anything is giving your own experience to a person who suffers.” (Participant B) “I feel like my friends’ counselor and people’s counselor, they always trust in me and I help them.” (Participant C)
	Significant relationships	^∗^I have role models that serve me as guidance and support *Tengo personas de referencia que me sirven de guía y apoyo.*	“Someone that helps you if you have a problem and you think you can’t solve it; someone that helps you to solve the problem and gives you motivation believing in yourself, and that’s why you have the strength to keep going until you solve it.” (Participant A) “Having an adult role model as mom and dad is essential for their development.” (Expert 4)
	Educative context	^∗^My values allow me a positive relation with my environment. *Mis valores me permiten una relación positiva con Mi entorno.*	“Having a good education. Education comes from home, if your parents educate you well on the street you will know how to behave, but if your parents don’t pay attention to you, probably other people will take you to the wrong path.” (Participant E) “It is convenient to raise the person in an integrate manner, as one that is immersed in the society, foment the holistic education. This means, more than a disciplinary development, a self-development and its environment.” (Expert 2)
	Cultural Integration	I feel integrated into the local culture and context. *Me siento integrado/a dentro de la cultura local.*	“There is some people that makes me feel bad. Some of the kids in my school…they always talking about my color. They say – you are chocolate color and we are white…what do you do in our country? – And that makes me feel bad, because not only in my country there are black people.” (Participant A) “There is a stigmatization because there’s a conjunction between the immigration and the youth. Prejudices are in both ways and it’s about to reconstruct and think …culture makes us different but the characteristic is that all of us are human beings …when the young people interact with others form different cultures, when they get to know each other they realize about their prejudices and reconstruct all those believes.” (Expert 1)
	Perception of available resources	In my context, there are services that represent a source of support for me. *En mi contexto existen servicios que son fuente de apoyo para mi*	“I think that the most difficult thing in life is not having a job…because without a job you don’t have money, and people without money can’t buy food, clothes, pay the rent, etc. …” (Participant A) “I think some resilience strategies are necessary, but with the crisis it’s limited. It’s clear that the psychosocial attention is necessary for parent and kids. The problem is that there are few professionals that work in public institutions as the CSMIJ and other organisms. And because of that there’s no efficiency in the service.” (Expert 3)
**Family Interaction**	Basic needs	I have good conditions of living that meet my needs. *Tengo una vivienda digna que cumple con mis necesidades.*	“Having a good place to live…hygienic and having food.” (Participant D) “To grow healthy it’s important a good nutrition at home” (Participant A)

*^∗^Items included in CYRM.*

Interviews from the second study highlighted the importance of self-awareness and the capacity to recognize and regulate one’s own emotions when managing difficult situations. The adult experts in particular noted that recognizing one’s own feelings and emotions caused by adversities is an important factor to cope with adversity. It enables the person to reflect over more effective alternatives and, thus, leading to problem solving. This finding reflects previous research by Troy and Mauss (2011), who found that emotional regulation is connected with resilience through two strategies: attention control and cognitive reappraisal. These findings also point to the need for further research on the benefits and the process of emotion regulation strategies that may help us to better understand resilience (Kay, 2016).

Another theme emerging from the qualitative data was the importance of having a good sense of humor. Humor was described as a resource to cope with problems and to relate with other people within one’s immediate environment (Cann and Collette, 2014). A good sense of humor has the function of creating positive emotions, which facilitates communication and releases tensions, while enabling social support (Fredrickson, 2000; Martin and Lefcourt, 2004). The adult experts also mentioned the importance of a sense of humor and laughter as a resource that enables overcoming problems with optimism (Hughes, 2008).

Likewise, having goals can be related with motivation to achieve one’s purpose in life. Zolkoski and Bullock (2012), for example, found that it is important to have goals and a positive perspective for the future despite going through moments of adversity. Similarly, the young people interviewed also expressed the importance of following through with the aim they want to achieve.

Another relevant theme for the young participants was adaptation: the capacity to adapt to conflicting situations (Masten and Tellegen, 2012). One participant stated that she tried to adapt to changing situations, that she reflected on these situations to learn from the experience. This is a relevant issue, not only for immigrants that live in cultural contexts, very different from their own, but to reveal the capacity young people have to adapt to changes or situations that are perceived as difficult.

Interaction with others is another subcategory contained in the CYRM-28. The establishment of significant relationships that work as a source of practical and emotional support and offer strength to solve problems, feeling loved and valued, are of great importance. Family attachment is the aspect that appeared most frequently. In addition, having a role model in the family helps young participants to learn how to solve problems. In this sense, a supporting family environment promotes adaptation and positive outcomes in children (Marici, 2015).

Regarding the capacity of looking for support, the participants revealed their ability to ask for help to external people, either institutions, other sources of help or people from their close environment. This is the ability to negotiate with their communities and others to find health-sustaining resources (Ungar, 2008).

One of the experts stated that some children tend to make agreements with their parents in order to take adequate actions. Young people consider it important to receive the approval from their parents about the actions they are taking. Another expert explained his personal experience with his daughters to strengthen their self-esteem as a protective factor associated with good psychosocial functioning (Kidd and Shahar, 2008), to enable them to cope in situations where they can be discriminated because of their physical and/or ethnic condition.

#### Reformulated Items

The experts considered most of the items as understandable. However, both the adult experts and the results of the interviews with youth, highlighted the need to reformulate items 1, 3, 9, 19, and 22.

The reformulated items were: (1): “I have people I look up to.” (*Conozco personas a las que admiro*), item reformulated (IR): “I know people who are an example to follow” (Conozco personas que son un ejemplo a seguir); (3): “Getting an education is important to me” (*Tener una educación es importante para mí*), IR: “Getting an academic education is important to me” (*Tener una educación académica es importante para mí*); (9): “Spiritual beliefs are a source of strength for me” (*Mi fe me da fuerzas*), IR: “I have faith and trust in me to achieve my goals” (*Tengo fe y confianza en mí para conseguir mis objetivos*); (19): “I am treated fairly in my community” (*Soy tratado con igualdad dentro de mi comunidad*), IR: “I feel that I am being treated equally by people around me despite differences in ethnicity, gender, religion, culture or beliefs” (*Siento que soy tratado con igualdad por las personas que me rodean a pesar de que haya diferencias de etnia, género, religión, cultura o creencias*); (22): “I participate in organized religious activities” (*Participo en diversas actividades religiosas*), IR: “I participate in activities outside the school” (sports, religious, artistic, volunteering etc.). (*Participo en actividades fuera de la escuela* (*deportivas, religiosas, artísticas, voluntariado* etc.).

Item 23, “I think it is important to help out in my community” was understood by almost all the participants, but, at the same time, was perceived as being irrelevant to the local context. From the results, we could see that the concept of community is not well established within the local context and may be related to item 22 “I participate in organized religious activities.” Accordingly, item 23 was erased. Relevance and comprehension was assessed by the advisory committee, together with reformulation of items included in the final version.

## Study III

The aim of the third study was to explore the psychometric properties of the CYRM-32 adapted to Spanish scale. Three hypotheses were proposed to assess the validity:

Hypothesis 1: Data will confirm the factor structure of the CYRM-32 adapted scale (3 factors).
Hypothesis 2: The CYRM-32 scale and its subscales will exhibit adequate psychometric properties (internal consistency Cronbach’s α, test–retest reliability and convergent and discriminant validity).
Hypothesis 3: There will be significant differences in youth scores on the CYRM-32 adapted depending on the gender, ethnicity and age of participants.

### Participants

A total of 432 young people participated in the study. All of them presented one of the following risk factors: having experienced a traumatic event, immigration, poverty, exposure to violence, substances abuse and risk of social exclusion. They were randomly selected from four schools situated in a vulnerable and economically deprived neighborhood, and concurrent users of especial educational supports, community programs and mental health services.

The average age of participants was 14.99 years (SD = 2.23), range 12–19; 45.1% were girls and 54.9% were boys; 61.5% were European, 15.6% Moroccan, 10% Latin American, 7.9% Asian and 4.8% belonged to different ethnic groups. All participants had been in Spain for more than 2 years and understood the language correctly.

### Instruments

#### CYRM-32 Spanish Version

It includes 32 items designed to assess resilience features in children and youth. It was adapted from the original scale CYRM-28, which was designed to explore the individual, relational community and cultural resources, available to youth 12 to 23 years. In Spanish adaptation, only three factors were found and their Cronbach’s α were: Family interaction (0.720), Interaction with others (0.506), Individual skills (0.622) and for the totality of scale (0.783). It is a self-reported measured where for each question participants use a 5-point Likert scale (1 = not at all to 5 = a lot).

#### Item “Are You Depressed or Sad?”

Item “Are you depressed or sad?” was used to assess the depression mood of the participants. This item was adapted from the “Are you depressed?” Screening for depression in the terminally ill (Chochinov et al., 1997) and it was evaluated through numeric scale of 0–10 (0-not depressed, 10-worst possible depression). A high sensitivity (1.00) and specificity (1.00) to identify depressed mood, and absence of false positive and negative rate (0.00) were presented.

#### Brief Resilient Coping Scale (BRCS)

This scale was composed by four items to assess strategies to cope with the stress in a highly adaptive manner (Limonero et al., 2014). Brief Resilient Coping Scale (BRCS) is a self-reported measured where for each question participants use a 5-point Likert scale where 1 “does not describe you at all” and 5 means “it describes you very well.” Higher scores means grater resilient coping. Cronbach’s α coefficient was 0.71 (Study 3: α = 0.72) and a Pearson correlation was used to assess the temporal stability (test–retest reliability) and it was 0.69 (6 weeks).

#### Coping Strategies for Adolescents (ACS)

This scale was used to assess youth coping strategies and was developed by Frydenberg and Lewis (1996) and Frydenberg (1997). This scale is an 80-item checklist and has 18 subscales each contain between 3 and 5 items. Respondents indicate the extent to which the coping activity described was used (1 – “doesn’t apply or don’t do it,” 2 – “used very little,” 3 – “used sometimes,” 4 – “used often” and 5 – “used a great deal”). In terms of consistency the mean score across the 18 scales is 0.70, while the mean reliability across all scales is 0.68 (Frydenberg, 1997). (Study 3: α = 0.71).

#### Self-Concept Form 5 (AF5)

Self-concept and self-esteem were related with resilience in the literature (Buckner et al., 2003; Kidd and Shahar, 2008; Wille et al., 2008; Hopkins et al., 2014). This scale was designed by García and Musitu (1999) and is based on the theoretical model of Shavelson et al. (1976). It is made up of 30 items that are rated on a 5-point Likert-type scale ranging from 1 (Never) to 5 (Always) (Shavelson et al., 1976; García and Musitu, 1999). The scale was compost by 5 dimensions and their Cronbach’s α were for each one: social self-concept (0.69), academic self-concept (0.88), emotional self-concept (0.73), family self-concept (0.76) and physical self-concept (0.74) and Cronbach’s α for the totality of scale was 0.810 (Study 3: range α 0.70 at 0.82; total 0.75).

### Data Analysis and Procedure

Using the International Test Commission in context, construction and adaptation areas (Carretero-Dios and Pérez, 2007). Instruments with more than 15% of the data missing were excluded (Davey and Savla, 2010).

Regarding factorial validity, the sample was randomly divided into two subgroups, one for the initial analyses (*n* = 226) and the other for cross-validation (*n* = 206). Then, two CFAs were performed with the first subsample to determine which model explained the factorial structure better (Model 1-CFA and Model 2-CFA).

In each of the two models, it was expected that each observable variable would load only on the factor it was intended to measure; that measurement error associated with these variables would be uncorrelated. Model 1-CFA was a hierarchical and thus it considered that all covariance between each of the first order factors would be explained by a higher-order factor. Model 2-CFA was a bi-factor model and thus it considered that all covariance between each of the first order factors would be explained by a general dimension which load on all items at the same as the factors as suggested by Konkolÿ et al. (2014).

Estimates indices were obtained using the maximum likelihood method. So as to assess model fit, absolute fit indexes (χ^2^, χ^2^/*df*), relative fit indexes (IFI) and non-centrality fit indexes (CFI, RMSEA, SRMR) were used, as well as criteria for acceptable fit based on the degree of adjustment described by Hair et al. (2010) (ratio χ^2^/*df* < 5; SRMR < 0.08; RMSEA < 0.08; GFI, CFI and IFI > 0.90). Finally, we conducted cross-validation analyses (CVA) with the model, which showed the best fit in order to explore the sample invariance of the model.

Reliability assessment was conducted using Cronbach’s α (Cronbach, 1951) for internal consistency and for the reproducibility the test–retest was carried on in two time points (*n* = 162) separated by approximately 2 months. Paired samples *t*-test were used to test differences in scores between these two administrations of the CYRM-32 adapted. The Pearson product moment correlation was used to establish construct validity and confirm hypothesis 2, describing the relationship between CYRM-32 scores and BRCS; Coping strategies for adolescents (ACS); Self-concept form 5 (AF5), and the item “Are you depressed or sad?” The floor or celling effect problems were also identified (Terwee et al., 2007).

In order to explore the functioning of resilience among different groups of participants (hypothesis 3) a multivariate analysis of variance (MANOVA) was carried on the study group sample, with gender, age and ethnicity (European, Gitano, Asiatic, African, Caribbean, Latin America, Nord America and Canada or others). The data were analyzed using SPSS v.20. and AMOS v.22.

### Results

#### Confirmatory Factor Analysis

**FIGURE 2:**
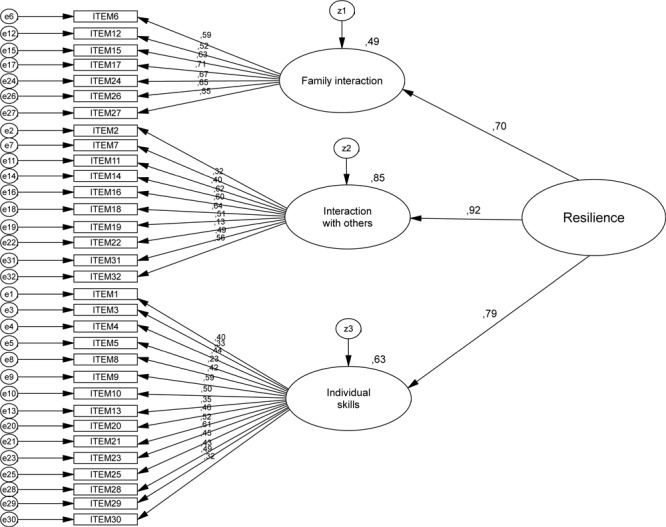
Standardized estimates, as well as the squared multiple correlations for Model 1-CFA.

We first tested the hierarchical model (Model 1-CFA) with the first subsample. Figure 2 shows the standardized estimates, as well as the squared multiple correlations (located at the top of each item). Table 3 shows Chi-square statistic was significant, probably due to the sample size (Hair et al., 2010). The ratio χ^2^/*df* = 1.83 < 5, the RMSEA < 0.08 and the SRMR < 0.08 were well inside the limits that allowed the model to be accepted. However, the rest of the fit indices fell short of the standard limits of acceptance and thus indicate that the model does not represent the data well.

**Table 3:** Goodness of fit statistics for the hierarchical confirmatory factor analysis (Model 1-CFA), the bi-factor confirmatory factor analysis (Model 2-CFA) and the bi-factor cross-validation analysis (Model 2-CVA).

	χ^2^	*df*	*p*	χ^2^/*df*	GFI	IFI	CFI	RMSEA	SRMR
Model 1-CFA *N* = 226	841.65	461	0.000	1.83	0.819	0.772	0.768	0.061	0.068
Model 2-CFA *N* = 226	741.63	433	0.000	1.71	0.838	0.819	0.812	0.056	0.060
Model 2-CVA (*N* = 226/206)	1502.58	866	0.000	1.73	0.826	0.815	0.808	0.041	0.060

*GFI = goodness of fit index; IFI = incremental fit index; CFI = comparative fit index; RMSEA = root mean square error of approximation; SRMR = standardized root mean square residual.*

**FIGURE 3:**
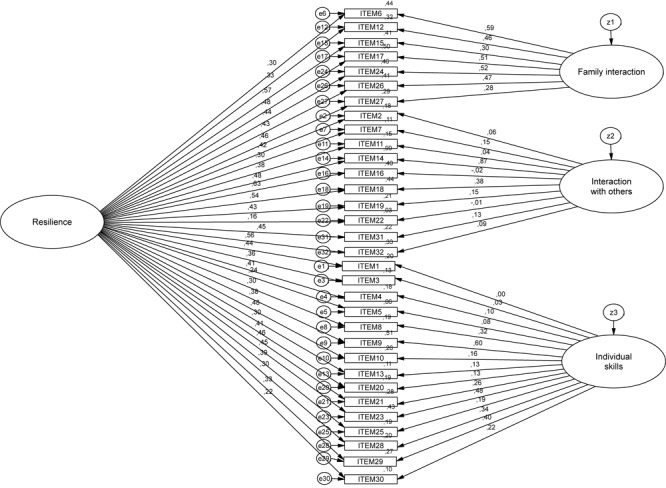
Standardized estimates, as well as the squared multiple correlations for Model 2-CFA.

We then tested the bi-factor model with the same first subsample. Figure 3 shows the standardized estimates, as well as the squared multiple correlations (located at the top of each item). Regarding fit statistics, Chi-square statistic was significant, again probably due to the sample size (Hair et al., 2010), the ratio χ^2^/*df* = 1.71 < 5 was well inside the limits that allowed the model to be accepted. The RMSEA < 0.08 and the SRMR < 0.08 were well inside the limits that allowed the model to be accepted. The remaining adjustment indexes were slightly better than for Model 1-CFA but fell slightly short of the standard limits of acceptance. So, in order to test the validity of the model, a CVA of Model 2 was carried-out (Model 2-CVA).

The fit statistics for Model 2-CVA (see Table 3) were similar to those of Model 1-CFA. However, the model comparison statistics carried out against the unrestricted model, establishing equality restrictions between groups for measurement weights (Δχ^2^ = 12.23, *p* = 0.42), structural covariance (Δχ^2^ = 31.44, *p* = 0.43), structural residuals (Δχ^2^ = 33.30, *p* = 0.39) and measurement residuals (Δχ^2^ = 53.62, *p* = 0.60), show that fit is not significantly reduced in relation to the model without restrictions, which means that the tested model works similarly in both samples.

#### Reliability Assessment and Floor and Ceiling Effects

Internal consistency reliability was estimated by Cronbach’s α for each of components and for the totality of the scale. The α’s were 0.792 (Family interaction), 0.715 (Interaction with others), 0.778 (Individual skills) and for the totality of the scale was 0.877, which demonstrated a consistent scale (see Table 4).

**Table 4:** CYRM-32 item’s scale and its three dimensions.

Dimensions and items	Cronbach’s α
**Family interaction** **6.** Mis padres o tutores lo saben todo sobre mí. (*My parent*(*s*)*/caregiver*(*s*) *know a lot about me* **12**. I talk to my family/caregiver(s) about how I feel. (*Hablo sobre cómo me siento con mi familia o tutores legales* **15.** I know where to go in my community to get help. (*Sé dónde acudir dentro de mi comunidad, cuando tengo algún problema* **17.** My family stands by me during difficult times. (*Mi familia me apoya en los momentos difíciles*) **24.** I feel safe when I am with my family/caregiver(s). (*Me siento a salvo junto a mis padres o tutores*) **26.** I enjoy my family’s/caregiver’s cultural and family traditions. (*Disfruto de las tradiciones familiares con mis padres o tutores* **27.** I enjoy my community’s traditions. (*Disfruto de las tradiciones de mi comunidad*)	0.792
**Interaction with others** **2.** My values allow me a positive relation with my environment. (*Mis valores me permiten una relación positiva con mi entorno*) **7.** If I am hungry, there is enough to eat. (*Si tengo hambre, siempre hay suficiente comida para alimentarme*) **11.** People think that I am fun to be with. (*La gente piensa que soy una persona divertida*) **14.** I feel supported by my friends. (*Mis amigos me apoyan*) **16.** I feel I belong at my school. (*Siento que formo parte de mi escuela*) **18.** My friends stand by me during difficult times. (*Mis amigos me apoyan en los momentos difíciles*) **19.** I feel that I am being treated equally by people around me despite differences in ethnicity, gender, religion, culture or beliefs. (*Siento que soy tratado con igualdad por las personas que me rodean a pesar de que hayan diferencias de etnia, género, religión, cultura o creencias*) **22.** I participate in activities outside the school (sports, religious, artistic, volunteering etc.). (*Participo en actividades fuera de la escuela* (*deportivas, religiosas, artísticas, voluntariado* etc.) **31.** I support my peers. (*Doy apoyo a mis compañeros*) **32.** I have role models that serve me as guidance and support. (*Tengo personas de referencia que me sirven de guía y apoyo*)	0.715
**Individual skills** **1.** I know people who are an example to follow. (*Conozco personas que son un ejemplo a seguir*) **3.** Getting an academic education is important to me. (*Tener una educación académica es importante para mí*) **4.** I know how to behave in different social situations. (*Sé comportarme teniendo en cuenta las normas sociales*) **5.** When I face a problem, I’m aware of my emotions and act according to how I feel in that moment. (*Ante algún problema soy consciente de mis emociones y actúo según como me siento en el momento*) **8.** I try to finish what I start. (*Intento finalizar todo lo que empiezo*) **9.** I have faith and trust in me to achieve my goals. (*Tengo fe y confianza en mí para conseguir mis objetivos*) **10.** Despite difficulties, I usually smile. I think that I have good sense of humor. (*A pesar de las dificultades suelo sonreír. Me considero una persona con buen sentido del humor*) **13.** I am able to solve problems without harming myself or others (for example by using drugs and/or being violent. (*Puedo solucionar mis problemas sin hacerme daño ni hacer daño a terceras personas* (*por ejemplo sin caer en adicciones como la droga y sin usar la violencia*) **20**. I have opportunities to show others that I am becoming an adult and can act responsibly. (*Puedo demostrar a los demás que soy una persona adulta y responsable*) **21.** I am aware of my own strengths. (*Soy consciente de mis puntos fuertes*) **23.** My strength helps me to go on and achieve my goals. (*Mi fortaleza me ayuda a seguir adelante y alcanzar mis objetivos*) **25.** I have opportunities to develop skills that will be useful later in life (like job skills and skills to care for others. (*Tengo la oportunidad de desarrollar habilidades que me serán útiles en el futuro* (*Habilidades relacionadas con un oficio y habilidades sociales*) **28.** I have aspirations and a clear and realistic vision of the future. (*Tengo aspiraciones y una visión de futuro clara y realista*) **29.** I usually make my own decisions and don’t let myself be influenced by others. (*Tiendo a tomar mis propias decisiones y no me dejo llevar por los demás*) **30.** I’m able to adapt to changes (*Soy capaz de adaptarme a los cambios*)	0.778 **0.877^∗^**

*^∗^ Cronbach’s α for totally of the scale.*

In a sample of 162 youth, a Pearson correlation was used to assess the temporal stability of the CYRM-32 over 2 months, yielded a value of 0.695 (*p* < 0.01) for the total score of the scale; and for each of the three factors (family interaction, interaction with others and individual skills) this correlation was: 0.784 (*p* < 0.01), 0.781 (*p* < 0.01), 0.787 (*p* < 0.01), respectively.

The highest (157) and lowest (72) score were obtained only once. In addition, none of the participants obtained the lowest and highest CYRM-32 total scores, focusing the absence of floor or ceiling effects problems.

#### Convergent and Discriminant Validity

Correlations between total score of the CYRM-32 and its three dimensions with four measures of mood, resilience, coping strategies, and self-concept were represented in Table 5. The CYRM-32 scale and all of its dimensions were positively correlated with resilience measure, self-concept (except the dimension emotional self-concept) and with thirteen of the eighteen coping strategies (the positive strategies), and negatively correlated with mood depressed or sad and four negative coping strategies: lack of coping, reduction of tension (using drugs), self-incriminating and reserve it for yourself.

**Table 5:** Correlations between dimensions of CYRM-32 and others measures.

Measure	Family interaction	Interaction with others	Individual skills	CYRM-32
Are you depress or sad?	–0.347**	–0.328**	–0.314**	–0.391**
Brief Resilient Coping Scale	0.270**	0.317**	0.389**	0.424**
**Coping strategies for adolescent**				
Search for social support	0.394**	0.353**	0.328**	0.425**
Concentrate in solving the problem	0.302**	0.380**	0.475**	0.473**
Strive and have success	0.352**	0.357**	0.463**	0.477**
To worry	0.215**	0.213**	0.313**	0.305**
To invest in intimate friends	0.171**	0.314**	0.240**	0.291**
Search release	0.244**	0.285**	0.264**	0.318**
Make illusions	0.024**	0.107**	0.098*	0.094
Lack of coping	–0.163**	–0.252**	–0.231**	–0.260**
Reduction of tension	–0.144**	–0.179**	–146^∗∗^	–0.185**
Social action	0.197**	0.069	0.099*	0.140**
Ignore the problem	–0.067	–0.064	–0.073	–0.083
Self-incriminating	–0.105*	–127^∗^	–0.095	–0.128**
Reserve it for yourself	–0.227**	–142^∗∗^	–0.86	–0.174**
Search spiritual support	0.222**	0.101*	0.082	0.154**
Focus on the positive	0.357**	0.362**	0.431**	0.464**
Search for professional help	0.357**	0.215**	0.233**	0.316**
Search relaxing amusements	0.190**	0.282**	0.265**	0.296**
Physical distraction	0.240**	0.356**	0.290**	0.350**
**Self-concept form 5**				
Social self-concept	0.209**	0.470**	0.364**	0.418**
Academic self-concept	0.305**	0.344**	0.346**	0.420**
Emotional self-concept	–0.014	0.098*	0.076	0.066
Family self-concept	0.486**	0.366**	0.314**	0.455**
Physical self-concept	0.161**	0.349**	0.339**	0.345**

*^∗∗^Correlation is significant at the *p* < 0.01 level (2-tailed). ^∗^Correlation is significant at the *p* < 0.05 level (2-tailed).*

Finally, a MANOVA was conducted to test our 3 hypothesis that significant differences exist between different ethnic, gender and age. No significant multivariate main effects were found for age *F*(71, 352) = 1,437, *p* < 0.05 or gender, *F*(72, 359) = 1,062, *p* > 0.05, but there was a significant main effect for ethnicity *F*(71, 358) = 1,714, *p* < 0.01. That suggest only partial support for our hypothesis, but shows that the different cultural aspect of the participants were connected with resilience process.

## Discussion

This article includes three studies that reflect the complexity of the development of a culturally adapted scale. The used of mixed methods allowed us to better understand the conflicting items that were found in the original scale and to deepen the interpretation of the resilient processes by developing new items specific to our culture.

The results obtained in the first and third study, showed that the initially Spanish version CYRM-28 and the last CYRM-32 have good psychometric properties. Despite these findings, qualitative assessment of cultural and contextual relevance, highlighted several items that required adaptation to the local context. These findings regarding variation in instrument items reflect the notion that culture and context shape the environment and consequently influence the resilience process of people living there. For this reason, including cultural diversity allows for a better understanding of the construct from a heterogeneous and socio-ecological perspective (Ungar, 2011).

The present work includes three studies (Figure 1). In the first study, the translated CYRM-28 was assessed for validity of its psychometric properties. The distribution of items was different and the factors did not correspond to all the three theoretical factors of the original scale. Additionally, the emerging factors were provided alternate names, as identified by an expert review panel.

The first factor, “Family interaction” explained 17.7% of the variance, and related to a broad concept of family. It includes eight items that have high loadings and strong internal consistency. The second factor, “Interaction with others” explained 7.17% of the variance and reflects social interaction across various contexts (such as peer group, school, and religious settings). All seven items had significant loadings but less internal consistency. The final factor, “Skills or individual resources,” explained 6% of variance, and included nine items, six of them with adequate saturation and loadings with medium internal consistency.

As observed in other studies that obtained similar results (Liebenberg et al., 2012; Daigneault et al., 2013), some items presented problematic factor loading. In our study, items 1, 3, 9 and 22 failed to exhibit strong factor loading on any of the three final components. Also, four items (2, 5, 10 and 28) were excluded due to inadequate factor loadings.

The divergence of the structure of the scale with the original version further emphasizes the cultural and contextual factors that differentiate populations, and how each culture understands the phenomenon of resilience (Ungar, 2004, 2008). These differences are probably due to the fact that the scale validation was conducted with youth in Canada (Liebenberg et al., 2012); a place where there was a difference in the risk context of participants: in Canada the sample included high-risk youth (users of services such as care of children, mental health, justice), whereas in our study, the participants were young people belonging to a neighborhood with high risk and vulnerability indicators. It is however, more probable that the variability is due to cultural and contextual differences. For example, the factor structure identified in New Zealand (Sanders et al., 2015) is also different from that of the factor structure identified in Canada (Liebenberg et al., 2012), while the sample in both sites was the same.

From qualitative data, nine items were developed and were included in the final version of CYRM.

The themes related with the Individual factor were self-awareness (“When I face a problem, I’m aware of my emotions and act according to how I feel in that moment”); Sense of humor (“Despite difficulties, I usually smile. I think that I have good sense of humor”); Vision of the future (“My strength helps me to go on and achieve my goals” and “I have aspirations and a clear and realistic vision of the future”); Adaptation (“I’m able to adapt to changes”); Autonomy (“I usually make my own decisions and don’t let myself be influenced by others”).

Three of the new themes related to items identified during the original development of the CYRM (Ungar and Liebenberg, 2011): Support for others (“I support my peers”); Significant relationships (“I have role models that serve me as guidance and support”); Education context (“My values allow me a positive relation with my environment”).

Only one new item related to basic needs, stemming from Family interaction (“I have good conditions of living that meet my needs”). This item was not included in the final version as it strongly reflected the original CYRM item “I have enough to eat.”

To increase their comprehension and relevance five items were reformulated (items 1, 3, 9, 19, 22).

Drawing on the qualitative data, two items were reformulated to better align with youth identified resilience factors. Because participants felt it was more important to believe in themselves that to have spiritual beliefs, item 9 “Spiritual beliefs are source of strength for me” was changed to “I have faith and trust I can achieve my goals.” Similarly, item 22 “I participate in organized religious activities” was reformulated to include other activities “I participate in activities outside the school (sports, religious, artistic, volunteering.” Here youth noted that young people often interact with their community by participating in extracurricular activities, especially sport, rather than religious activities (see also Moreno et al., 2016).

Our findings show that the Spanish CYRM-32 has good psychometric properties and highlight the importance of the process of prior cultural adaptation of the scale. Two of three hypotheses of the study were confirmed except for the hypotheses three, where only differences in the CYRM-32 scores were found in ethnicity.

With respect to the first hypothesis, the three-factor structure was confirmed, which is consistent with the original CYRM scale (Ungar, 2008; Ungar and Liebenberg, 2009) and with its adaptation among French Canadian youth (Daigneault et al., 2013). The fact that a bi-factor model as the one proposed by Konkolÿ et al. (2014) fitted our data better than a hierarchical model implies that people showing high resilience in an individual dimension, tends to show also high resilience in the dimensions family interaction and interaction with others and also supports the idea of calculating a single overall resilience score in addition to subscales for each of its dimensions (Konkolÿ et al., 2014).

The scale CYRM 32 and its three components: family interaction, interaction with others and individual skills, presented strong internal consistencies, as reveled by high values of Cronbach’s α, consistent with other’s CYRM-28 scales validation (Liebenberg et al., 2012; Daigneault et al., 2013; Sanders et al., 2015) and with higher values than those obtained in the cultural adaptation process. Furthermore, no participant scored the highest and lowest score of 32, so no floor or ceiling effects were detected (Terwee et al., 2007). Temporal stability of scores over 2 months was also observed in the CYRM-32’s total scale and its three dimensions, confirming thus the second hypothesis.

As hypothesized, the results also confirm that CYRM-32 and its three factors, were positively and significantly correlated with measures of resilience (BRCS), Self-Concept form 5 (except emotional self-concept) and thirteen positive coping strategies for adolescent: search for social support, concentrate in solving the problem, strive and have success, to worry, to invest in intimate friends, search release, make illusions, social action search spiritual support, focus on the positive, search for professional help, search relaxing amusements and physical distraction; while showing a negative and significant correlation with four negative coping strategies: lack of coping, reduction of tension (using drugs), self-incriminating and reserve it for yourself; also with the item “Are you depressed or sad” in concordance with the authors who defined resilience how “good mental health” (Klasen et al., 2010; Williams and Nelson-Gardell, 2012; Collishaw et al., 2016). Despite some research show the relationship between emotion and resilience (Troy and Mauss, 2011; Kay, 2016) the emotional self-concept was not significant correlated with CYRM-32. In spite of this, all other correlations provide evidence of the criterion-related validity of CYRM-32.

In regard to the hypothesis tree, where we were expected to find significant differences in youth scores depending on the gender, ethnicity and age of participants, we only found significant differences with ethnicity in concordance with other validation of CYRM-28 such as the one undertaken in New Zealand (Sanders et al., 2015) and in Canada with French speaking youth (Daigneault et al., 2013). Despite the significant main effect that was found for gender in the Canadian validation, the key differences were observed in ethnicity between visible minority youth and visible majority youth (Liebenberg et al., 2012). These findings support the idea that the developed of resilience is influenced by culture and context as others researches (Masten and Coatsworth, 1998; Ungar, 2004, 2011; Clauss-Ehlers, 2008).

The adaptation and validation of this scale using mixed-methods could be used in Spain to evaluate the effectiveness of future interventions projects to promote resilience among children and youth at risk (Liebenberg et al., 2012).

This finding has implications for the assessment practices and interventions for all professionals who work with youth in context of risk.

## Study Limitations and Strengths

The Spanish version of the CYRM-32 is an adequate and validity tool to measure resilience, which will permit to assess the effectiveness of future interventions to promote resilience among children and youth at risk (Liebenberg et al., 2012) and it covers a gap regarding the existence of validated instruments to measure resilience in this population.

Different samples of at risk youth were represented in the groups of participants. They were randomly selected. Furthermore, persons from different ethnic groups were included in the final sample. Concerning limitations of the study, the items of the scale were all phrased positively and that might have decreased the reliability of the CYRM-32. However, the results show a high internal consistency.

In future research, the predictive validity of the CYRM-32 (Terwee et al., 2007), and the scale’s sensitivity to change over the course of intervention would need to be established.

Despite these limitations, the Spanish version of the CYRM-32 presents good psychometric properties and could be an alternative tool to measure resilience in Spanish-speaking at risk youth.

## Data Availability

All relevant data is contained within the manuscript. All datasets generated/analyzed for this study are included in the manuscript and the supplementary files.

## Ethics Statement

The Ethics Committee Institutional Review Board of the Consorci Sanitari de Terrassa (Spain) was approved the study. In all three studies, the written informed consent was obtained prior to data collection, from all adult participants and from the parents/legal guardians of all non-adult participants, as well as the directors of secondary schools who collaborated in the research.

## Author Contributions

ML, TGR, and JTL contributed in the conceptualization, design, methodology, results and discussion. ML led the Study I and Study III and wrote the first draft of the article. TGR supervised and integrated all revisions and formal aspects, and performed the submission. RRR performed part of the statistical analyses of Study III. LL provided important guidance as the author of the original scale and supervised the mixed methodology. ÁB carried out the empirical part of Study II. JGB supervised and reviewed the statistical analyses of Study III. All the authors revised and approved the final version of the manuscript.

## Conflict of Interest Statement

The authors declare that the research was conducted in the absence of any commercial or financial relationships that could be construed as a potential conflict of interest.
